# Polymorphisms of the Peroxisome Proliferator-Activated Receptor-γ (rs1801282) and its coactivator-1 (rs8192673) are associated with obesity indexes in subjects with type 2 diabetes mellitus

**DOI:** 10.1186/s12933-015-0197-0

**Published:** 2015-04-28

**Authors:** Peter Kruzliak, Andreana P Haley, Jovana Nikolajevic Starcevic, Ludovit Gaspar, Daniel Petrovic

**Affiliations:** Department of Cardiovascular Diseases, International Clinical Research Center, St Anne’s University Hospital and Masaryk University, Brno, Czech Republic; Department of Psychology, The University of Texas, Austin, TX USA; University of Texas Imaging Research Center, Austin, TX USA; Institute of Histology and Embryology, Faculty of Medicine, University of Ljubljana, Ljubljana, Slovenia; 2nd Department of Internal Medicine, University Hospital and Comenius University, Bratislava, Slovak Republic

**Keywords:** Peroxisome Proliferator-Activated Receptor-γ, Peroxisome Proliferator-Activated Receptor-γ Coactivator-1, Gene polymorphism, Association study, Type 2 diabetes mellitus, Obesity indexes

## Abstract

**ᅟ:**

The aim of this study was to clarify whether common single nucleotide polymorphisms (SNPs) of the Peroxisome Proliferator-Activated Receptor-γ (PPAR-γ) gene (rs1801282) and the Peroxisome Proliferator-Activated Receptor-γ Coactivator-1 (PGC-1α) gene (rs8192673) are associated with obesity indexes (BMI, waist circumference) in subjects with type 2 diabetes mellitus (T2DM) in Caucasian population. The second aim was to find an association of both polymorphisms with T2DM.

**Methods:**

Two exonic SNPs of both genes rs1801282 of the PPAR-γ gene and rs8192673 of the PGC-1α gene) were genotyped in 881 unrelated Slovene subjects (Caucasians) with T2DM and in 348 subjects without T2DM (control subjects).

**Results:**

Female homozygotes with the CC genotype of the rs8192673 had higher waist circumference in comparison with subjects with other genotypes. Homozygotes (females, males) with wild allele (Pro) of the rs1801282 (Pro12Ala polymorphism) had higher waist circumference in comparison with subjects with other genotypes. In the study, there were no differences in the distributions of the rs8192673 and the rs1801282 genotypes between patients with T2DM and controls. Linear regression analyses for both polymorphisms were performed and demonstrated an independent effect of the rs1801282 of the *PPAR*-*γ* on waist circumference in subjects with T2DM, whereas an independent effect on waist circumference was not demonstrated for the rs8192673 of the *PGC*-*1α* gene.

**Conclusions:**

In a large sample of the Caucasians the rs8192673 of the *PGC*-*1α* gene and the rs1801282 of the *PPAR*-*γ* gene were associated with waist circumference in subjects with T2DM.

## Introduction

Diabetes mellitus (DM) is a multifactorial metabolic disorder, characterized by hyperglycemia, resulting from abnormalities in insulin secretion, insulin action, or both [1]. Diabetes prevalence has increased dramatically in many countries over the past decades. The number of people with diabetes exceeds 300 million worldwide, most of them being patients with type 2 diabetes mellitus (T2DM) and it has become one of the most common chronic diseases in the world. There are predictions, based on a large number of studies, which indicate a growing burden of diabetes, particularly in the developing countries [2-4].

There are several pathogenic mechanisms that influence the development of diabetes and its complications [5-14]. T2DM is characterised by insulin resistance, dysfunction of pancreatic beta cells and enhanced hepatic gluconeogenesis. However, the exact pathophysiology of T2DM is still unknown. Abdominal obesity, sedentary lifestyle, advancing age, genetic and epigenetic factors affecting glucose homeostasis are thought to be key contributing factors [15,16].

Peroxisome proliferator-activated receptor-γ coactivator-1 (*PGC*-*1α*) gene is thought to be involved in three pathophysiological hallmarks of T2DM: insulin sensitivity, insulin secretion and hepatic gluconeogenesis [17,18]. Thus, functional sequence substitutions in *PGC*-*1α* may play an important role in the development of T2DM.

Results of the association studies involving the rs8192673 of the *PGC*-*1α* gene and the rs1801282 of the *PPAR*-*γ* gene published so far, reported different findings, showing either an association with T2DM risk or no association [16,18-22]. Moreover, the rs8192673 of the *PGC*-*1α* gene was reported to be associated with insulin resistance, obesity indices in women and with lipid metabolism and insulin secretion [21,23,24].

Both the PPAR-γ gene and the *PGC*-*1α* gene are potential candidates for modifying the risk of T2DM [6,8,25].

The aim of this study was to clarify whether common single nucleotide polymorphisms of the PPAR-γ gene (rs1801282) and the PGC-1α gene (rs8192673) are associated with obesity indexes (BMI, waist circumference) in subjects with T2DM in Caucasian population. The second aim was to find an association of both polymorphisms with T2DM.

## Materials and methods

In this cross-sectional study, 881 consecutive patients with T2DM from the outpatients clinics for patients with diabetes from central and north-eastern regions of Slovenia were enrolled as well as 348 subjects without diabetes. The research protocol was approved by The National Medical Ethics Committee. All the subjects enrolled in the study were Slovenian and were not related. Patients were classified as having type 2 diabetes according to the current report of the American Diabetes Association [1]. After the informed consent was obtained from the patient and subjects, a detailed interview was made concerning smoking habits, the duration and treatment of diabetes, arterial hypertension, and hyperlipidemia. Patients were asked whether they were smokers at the time of recruitment (“current smoker”). The body mass index (BMI) was calculated as weight in kilograms divided by the height in square meters. Obesity was determined defined as body mass index ≥ 30 kg/ m^2^ [26].

Systolic blood pressure (SBP) and diastolic blood pressure (DBP) in the right upper arm of the patients were measured while they were sitting (2 consecutive measurements). Subjects with T2DM with systolic blood pressure ≥ 140 mm Hg or diastolic blood pressure ≥ 85 mm Hg and/or subjects who were using antihypertensive drugs were considered to be hypertensive [27]. Secondary causes of arterial hypertension were excluded according to normal clinical exam (no systolic murmur above renal arteries) and normal serum electrolytes (exclusion of renal failure and hyperaldosteronism) [27].

Genomic DNA was extracted from 100 μl of whole blood using a Qiagen isolation kit. Genotypes of *PPAR*-*γ* were determined as described previously [28].

Blood samples for biochemical analyses: total cholesterol, triglyceride levels, high-density lipoprotein (HDL), low density lipoprotein (LDL) level and fasting blood glucose were collected after an overnight fasting. All blood biochemical analyses were determined by standard biochemical methods in the hospital’s accredited lab.

Data are expressed as means ± standard deviations or frequencies (percentages). The chi-square test was used to compare discrete variables. Continuous clinical data were compared by an unpaired Student’s t test (normal distribution by Kolmogorov Smirnov test) or by Mann–Whitney U test (for variables without normal distribution by Kolmogorov Smirnov test) or analysis of variance. A p < 0.05 was considered statistically significant. A statistical analysis was performed using the SPSS program for Windows version 20 (SPSS Inc. Illinois).

Continuous variables were expressed as means ± standard deviations when normally distributed, and as median (interquartile range) when asymmetrically distributed. Normality was tested with the Kolmogorov–Smirnov test. Continuous clinical data were compared by an unpaired Student’s *t* test or analysis of variance (ANOVA) when normally distributed, and Mann–Whitney U-test or the Kruskal-Wallis H-test when asymmetrically distributed. The Pearson ×^2^ test was used to compare discrete variables and to test whether the genotypes distributions are in Hardy-Weinberg equilibrium.

Pearson’s correlation was performed to examine the association between independent variables. Due to the high correlation of LDL cholesterol with total cholesterol (r = 0.86, p < 0.001) they were not included together in the same statistical model.

For determination of variables independently associated with waist circumference multivariate linear regression analyses were performed for both polymorphisms. Candidate variables to enter the models were the following: age, gender, plasma levels of LDL cholesterol and triglycerides, hypolipemic therapy, diabetes therapy, HbA1c and genotypes of the PPAR-γ (rs1801282) and the PGC-1α (rs8192673) polymorphisms. The results were presented as standardized ß coefficients and P-values for linear regression and by odds ratios and 95% CIs for logistic regression analysis.

A two-tailed P value less than 0.05 was considered statistically significant. A statistical analysis was performed using the SPSS program for Windows version 20 (SPSS Inc., Chicago, IL).

## Results

Clinical characteristics of the cases (patients with T2DM) and controls (subjects without T2DM) are summarized in Table 1. Cases had higher frequency of arterial hypertension, obesity (defined as body mass index ≥ 30 kg/ m^2^) in comparison with controls (Table 1). The values of body mass index (BMI), glucose, high sensitive C reactive protein (hsCRP), lipid parameters, fibrinogen levels were higher in subjects with T2DM. Additionally they had a higher frequency of hypolipemic therapy with higher values of triglycerides. On the contrary, total cholesterol, LDL cholesterol, and HDL cholesterol levels were higher in control subjects, as well as the percentage of current smokers. Results show no statistical significant differences in age and blood pressure values between cases and controls (Table 1).

**Table 1:** **Characteristics of subjects with type 2 diabetes (cases) and in subjects without diabetes (controls)**

**Characteristics**	**Cases**	**Controls**	**P**
Number	881	348	-
Age (years)	63.2 ± 10.3	61.3 ± 12.9	0.1
Male sex (%)	458 (52.0)	173 (49.7)	0.3
Duration of diabetes (years)	14.8 ± 8.9	-	-
Incidence of arterial hypertension (%)	458 (52.0)	103 (29.6)	<0.001
Systolic blood pressure (mm Hg)	148.9 ± 28.5	146.7 ± 22.7	0.06
Diastolic blood pressure (mm Hg)	84.9 ± 12.2	83.5 ± 11.8	0.3
BMI (kg/m^2^)^1^	30.4 ± 4.2	28.5 ± 3.5	<0.001
Obesity^2^	436 (49.5)	87 (25.0)	<0.001
Smokers (%)	61 (6.9)	45 (12.9)	<0.001
Glucose (mmol/l)	8.32 ± 2.52	5.18 ± 0.87	<0.001
Hba1c^3^	8.07 ± 1.54	-	-
hsCRP (mg/l)^4^*	2.1 (1.0-4.1)	1.1 (0.6-2.7)	<0.001
Fibrinogen (g/l)	4.10 ± 1.15	3.76 ± 0.83	<0.001
Hypolipemic drugs	698 (79.2)	66 (19.0)	<0.001
Total cholesterol (mmol/l)	5.5 ± 1.4	5.9 ± 1.6	<0.001
HDL cholesterol (mmol/l)	1.1 ± 0.3	1.3 ± 0.4	<0.001
LDL cholesterol (mmol/l)	2.6 ± 0.9	3.0 ± 1.1	<0.001
Triglycerides (mmol/l)*	1.9 (1.2-2.7)	1.3 (0.9-1.9)	<0.001

Continuous variables were expressed as means ± standard deviations when normally distributed and as median (interquartile range) when asymmetrically distributed.

^1^BMI – body mass index, ^2^obesity is defined as BMI ≥ 30 kg/m^2^, ^3^Hba1c – glycated hemoglobin A1c, ^4^hsCRP – high sensitive C reactive protein, *Continuous variables with asymmetric distribution.

The genotypes of both polymorphisms were in Hardy–Weinberg equilibrium (rs1801282 cases: ×^2^ = 3.22, p = 0.07; controls: ×^2^ = 0.56, p = 0.45; rs1801282 cases: ×^2^ = 0.07, p = 0.79; controls: ×^2^ = 2.5, p = 0.11).

The distribution of *PPAR*-*γ* gene (rs1801282) and the *PGC*-*1α* gene (rs8192673) among patients with T2DM and control subjects without T2DM is shown in Table 2. There were no differences in the distributions of the rs8192673 and the rs1801282 genotypes between patients with T2DM and control subjects without T2DM (Table 2).

**Table 2:** **Genotype distribution and allele frequencies of the rs8192673 of the** ***PGC*** **-** ***1α*** **gene and rs1801282 of the** ***PPAR*** **-** ***γ*** **gene in subjects with T2DM and in control subjects**

**Polymorphism**	**T2DM n = 881**	**Controls n = 348**	**p**
**rs8192673**			
TT	467 (53.0)	161 (46.3)	
TC	334 (37.9)	147 (42.2)	0.08
CC	80 (9.1)	40 (11.5)	
Allele frequencies			
T	1268 (71.9)	469 (67.4)	0.02
C	494 (28.1)	227 (32.6)	
**rs1801282**			
CC	631 (71.6)	250 (71.8)	0.50
CG	228 (25.9)	85 (24.5)	
GG	22 (2.5)	13 (3.7)	
Allele frequencies			
C	1490 (84.6)	585 (84.1)	0.06
G	272 (15.4)	111 (15.9)	

Results were presented as frequency (percentage).

**Figure 1:**
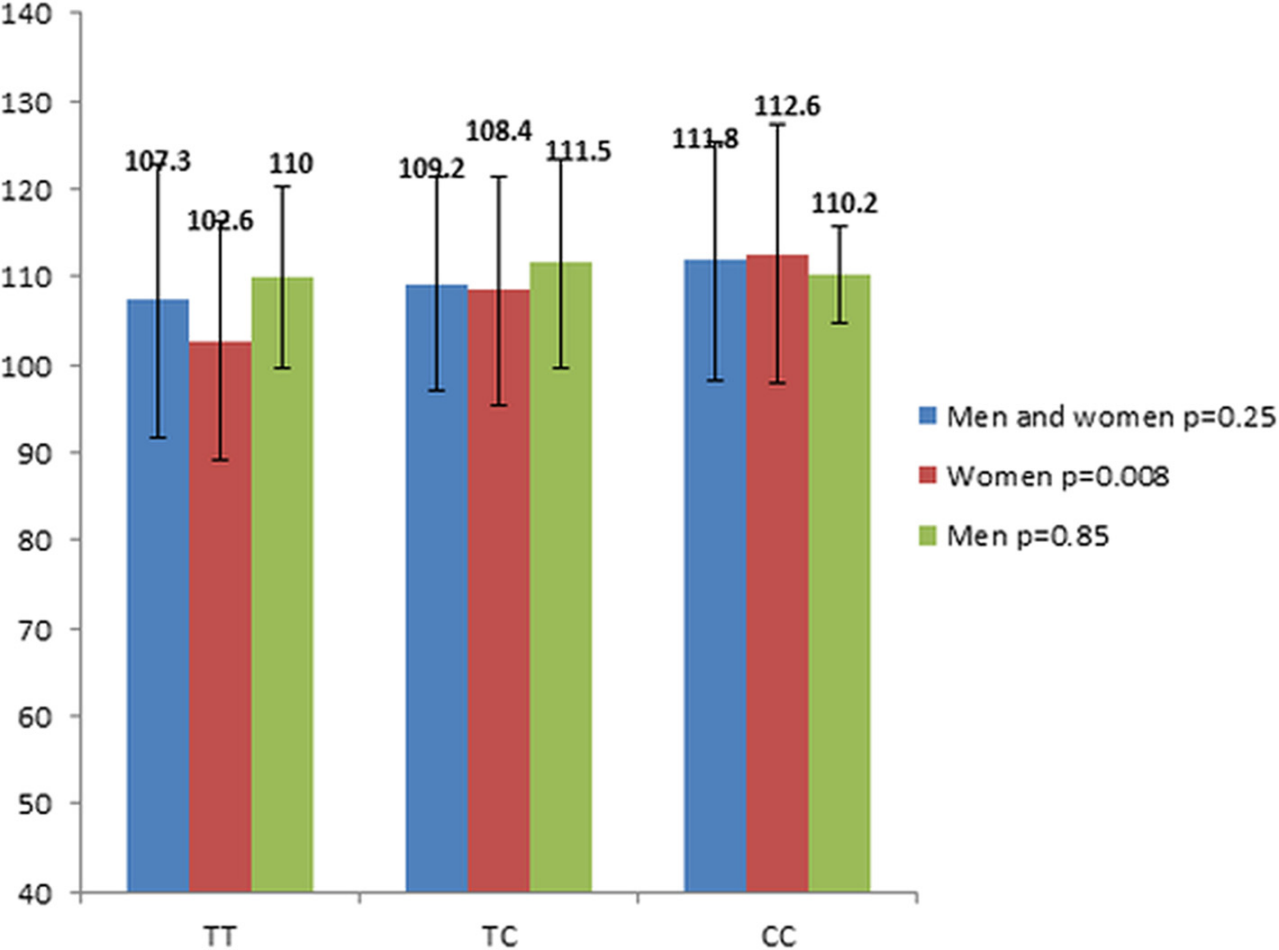
Waist circumference (cm) with regard to rs8192673 genotypes of the *PGC*-*1α* gene.

**Figure 2:**
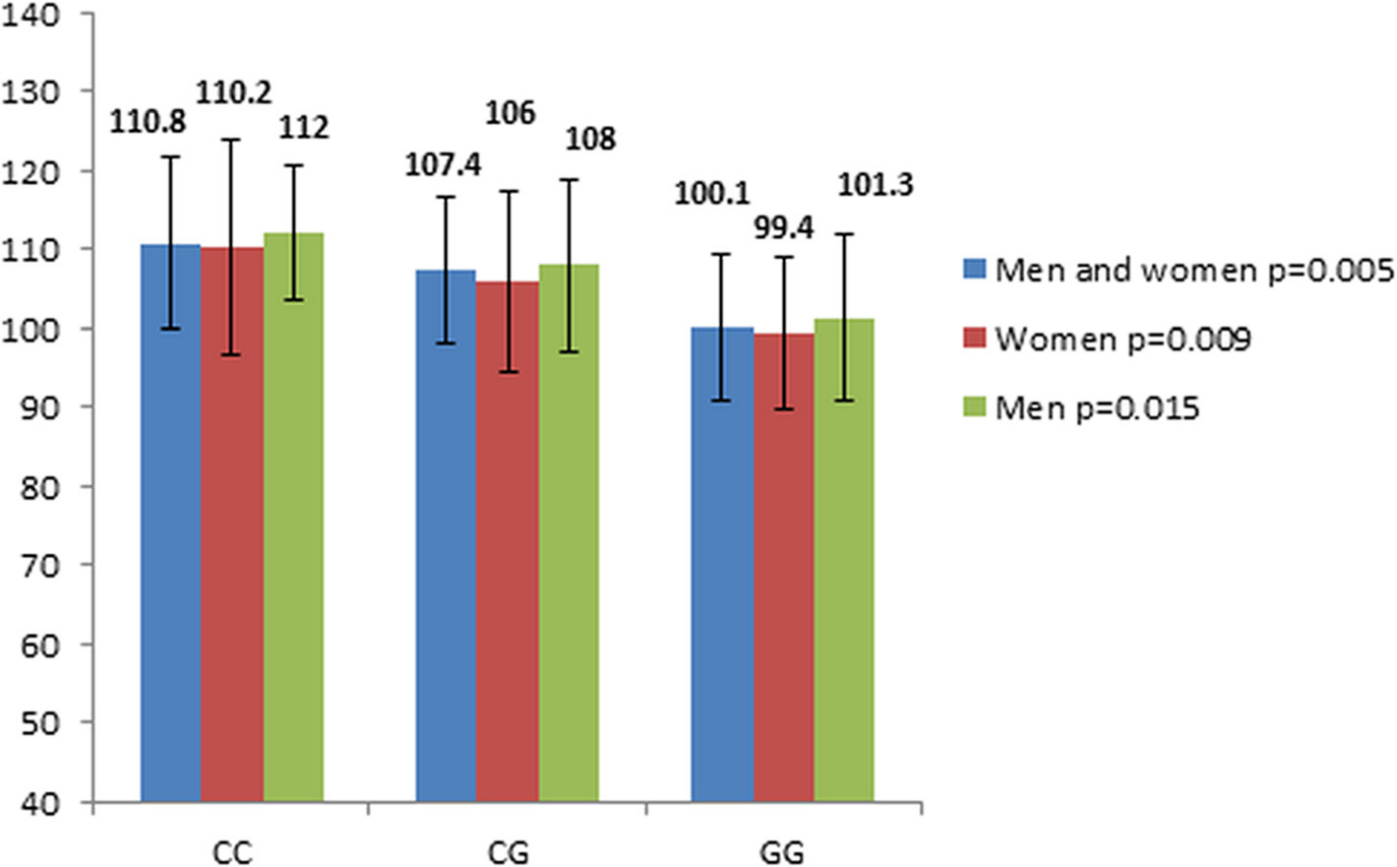
Waist circumference (cm) with regard to rs1801282 genotypes of the *PPAR*-*γ* gene.

The rs8192673 polymorphism was associated with waist circumference in subjects with T2DM. Specifically, female homozygotes with mutated allele (CC genotype) had higher waist circumference in comparison with subjects with other genotypes (Figure 1). Moreover, the rs1801282 (Pro12Ala polymorphism) polymorphism was associated with waist circumference in subjects with T2DM. Namely, subjects with the CC genotype (men and women, men, women) had higher waist circumference in comparison with subjects with other genotypes (Figure 2).

Linear regression analyses for both polymorphisms were performed and demonstrated an independent effect of the rs1801282 of the *PPAR*-*γ* on waist circumference in subjects with T2DM, whereas an independent effect on waist circumference was not demonstrated for the rs8192673 of the *PGC*-*1α* gene (Table 3).

**Table 3:** **Multivariate linear regression analyses for variables independently associated with waist circumference for both polymorphisms (rs8192673 of the** ***PGC*** **-** ***1α*** **gene, rs1801282 of the** ***PPAR*** **-** ***γ*** **)**

**rs8192673 of the** ***PGC*** **-** ***1α*** **gene**		
	**β**	**p**
Age (year)	−0.09	**<0.001**
Gender (0 = male, 1 = female)	−1.179	**0.019**
Triglycerides (mmol/L)	0.684	**<0.001**
LDL cholesterol (mmol/L)	0.288	0.759
HbA1c (%)	0.013	0.957
Insulin therapy (0 = no, 1 = yes)	−0.864	0.101
Hypolipemic therapy (0 = no, 1 = yes)	−0.479	0.786
TC*	0.537	0.271
CC*	0.646	0.54
**rs1801282 of the** ***PPAR*** **-** ***γ***		
Age (year)	−0.007	0.807
Gender (0 = male, 1 = female)	−1.213	**0.016**
Triglycerides (mmol/L)	−0.698	**<0.001**
LDL cholesterol (mmol/L)	−0.269	0.759
HbA1c (%)	0.134	0.954
Insulin therapy (0 = no, 1 = yes)	−0.812	0.122
Hypolipemic therapy (0 = no, 1 = yes)	0.550	0.755
CG**	−0.673	**0.024**
GG**	−0.832	**0.035**

*Reference genotype was TT; **Reference genotype was CC.

## Discussion

In this cross-sectional association study that involved more than one thousand Caucasians, the rs8192673 of the *PGC*-*1α* gene and the rs1801282 of the *PPAR*-*γ* gene failed to be associated with T2DM. These findings differ from an early report in the Slovene population from 2004 [28] and from the report from another Caucasian population [18], in which the authors reported a link between the rs8192673 of the *PGC*-*1α* gene and T2DM. The present finding in the Slovene population is in accordance with few other reports in the Caucasians [16,20], the Japanese population [21] and the Pima Indians [22], which also failed to demonstrate an association with T2DM. Moreover, rs1801282 (Pro12Ala polymorphism) of the *PPAR*-*γ* was also implicated in several studies to affect the T2DM risk [29]. Mekhani and co-workers reported that in the Iranian population there was a lower frequency of Ala allele in subjects with T2DM when these were compared to control subjects without T2DM (5.9% vs. 9.4%, p = 0.005). Besides being associated with a decreased risk of T2DM, this polymorphism was also associated with greater insulin sensitivity [28]. In the present study carried out in the Slovene population with T2DM there was no statistically significant difference in allele distribution, the frequency of the Ala allele was 15.4% and 15.9% in subjects with T2DM and in controls, respectively.

Due to some reports of an association of both polymorphisms (rs8192673, rs1801282) with obesity indexes [18,24,29], the influence of both polymorphisms on obesity indexes (BMI, waist circumference) in subjects with T2DM was also examined. In the present study in Slovene subjects with T2DM, the homozygous females with wild alleles had lower waist circumference in comparison with homozygous females with mutated alleles, whereas the effect of the rs8192673 genotypes was not demonstrated in males with T2DM. This finding is in accordance with an early report by Esterbauer and co-workers on general population [24]. They reported that wild allele was associated with lower waist circumference in middle-aged women, whereas there was no association between rs8192673 alleles in middle-aged men. Contrary to these reports, Ek and co-workers failed to report an association with another obesity index, namely BMI in their male/female type 2 diabetes and control populations [18,24]. In the present study, the rs1801282 (Pro12Ala polymorphism) polymorphism was also associated with waist circumference in subjects with T2DM. Namely, homozygotes (females, males) with wild allele (Pro) had higher waist circumference in comparison with subjects with other genotypes. Moreover, linear regression analyses for both polymorphisms were performed and demonstrated an independent effect of the rs1801282 of the *PPAR*-*γ* on waist circumference in subjects with T2DM, whereas an independent effect on waist circumference was not demonstrated for the rs8192673 of the *PGC*-*1α* gene. Contrary to our study, the Pro12Ala polymorphism did not show a significant effect on anthropometric and biochemical parameters in Iranian subjects with T2DM [29].

The exact mechanism of both polymorphisms is not completely clear, but they are expected to have various effects including affecting the insulin sensitivity of peripheral organs [11,23]. However, despite being associated with some obesity markers in this study and in several other studies, the influence of both polymorphisms is not strong enough to be associated with increased risk of T2DM [18,24,29]. Interactions among genetic (gene variants) and/or clinical factors (physical inactivity, diet, obesity…), which may have stronger effects in combination, are expected to increase the power of risk prediction of multifactorial disorders (including T2DM) and advance our understanding of the underlying biology of multifactorial disorders [2,3,30,31].

Beside these two polymorphisms of the *PGC*-*1α* and the *PPAR*-*γ* genes (rs8192673 and rs1801282) several other genes have been so far implicated by linkage analysis, association studies, and genome-wide association studies in the development of obesity and obesity-associated phenotypes [32-38]. Proteasome modulator 9 (PSMD9) gene was reported to be responsible for the linkage to obesity and obesity-associated phenotypes (waist circumference, overweight status) at the locus 12q24 [33]. Frayling and co-workers showed for the first time the contribution of the SNP rs9939609 in fat mass and obesity-associated gene FTO on excess of weight [37]. Common genetic variants of FTO-rs9939609 have positive associations with BMI and rs17782313 of the melanocortin 4 receptor (MC4R) gene with neck circumference in women [32]. Mutation in MC4R gene was found to be associated with morbid obesity in human beings [38]. Recently the association between the Fas apoptotic inhibitory molecule 2 (FAIM2)-rs7138803 polymorphism and greater obesity risk has been replicated [35]. Moreover, rs6232 of the proprotein convertase subtilisin/kexin-type 1 (PCSK1) was associated BMI [34]. Several different mechanisms of action have been proposed [32-36]. PCSK1 was found to activate precursors pro-opiomelanocortin (POMC), proinsulin and prorenin (citat). Similarly, since PSMD9 is a coactivator of insulin gene transcription, and in pancreatic overexpression of transgenic mice cause diabetes, PSMD9 variants may contribute to T2DM as well as to obesity [33,39,40]. Moreover, miRNA-dependent regulation of fat distribution by miR-196a2 and miR-1908-dependent regulation of lipid metabolism has recently been reported [36]. Additionally, according to the study of Corella and co-workers epigenetic factors may also be involved in the development of obesity [35].

Our study has few limitations, one of them being the cross-sectional design of the study. One of the major limitations of the current study is the absence of a replication study with independent samples. Another weakness is a relatively limited sample size. However, all the participants were enrolled from an ethnically homogenous population. Thus further studies are needed to replicate our findings in different ethnic groups with a larger sample size.

In conclusion, in a large sample of the Caucasians the rs8192673 of the *PGC*-*1α* gene and the rs1801282 of the *PPAR*-*γ* gene were associated with waist circumference in subjects with T2DM.
